# Spin relaxation in a single-electron graphene quantum dot

**DOI:** 10.1038/s41467-022-31231-5

**Published:** 2022-06-25

**Authors:** L. Banszerus, K. Hecker, S. Möller, E. Icking, K. Watanabe, T. Taniguchi, C. Volk, C. Stampfer

**Affiliations:** 1 JARA-FIT and 2nd Institute of Physics, RWTH Aachen University, Aachen, Germany; 2 Peter Grünberg Institute (PGI-9), Forschungszentrum Jülich, Jülich, Germany; 3 Research Center for Functional Materials, National Institute for Materials Science, Tsukuba, Japan; 4 International Center for Materials Nanoarchitectonics, National Institute for Materials Science, Tsukuba, Japan

**Keywords:** Electronic properties and devices, Quantum information

## Abstract

The relaxation time of a single-electron spin is an important parameter for solid-state spin qubits, as it directly limits the lifetime of the encoded information. Thanks to the low spin-orbit interaction and low hyperfine coupling, graphene and bilayer graphene (BLG) have long been considered promising platforms for spin qubits. Only recently, it has become possible to control single-electrons in BLG quantum dots (QDs) and to understand their spin-valley texture, while the relaxation dynamics have remained mostly unexplored. Here, we report spin relaxation times (*T*_1_) of single-electron states in BLG QDs. Using pulsed-gate spectroscopy, we extract relaxation times exceeding 200 *μ*s at a magnetic field of 1.9 T. The *T*_1_ values show a strong dependence on the spin splitting, promising even longer *T*_1_ at lower magnetic fields, where our measurements are limited by the signal-to-noise ratio. The relaxation times are more than two orders of magnitude larger than those previously reported for carbon-based QDs, suggesting that graphene is a potentially promising host material for scalable spin qubits.

## Abstract (web summary)

Graphene has long been considered to be a promising host for spin qubits, however a demonstration of long spin relaxation times for a potential qubit has been lacking. Here, the authors report the electrical measurement of the single-electron spin relaxation time exceeding 200 *μ*s in a bilayer graphene quantum dot.

## Introduction

The concept proposed by Loss and DiVincenzo to encode quantum information in spin states of QDs^1^ has laid the foundation of spin-based solid-state quantum computation. Spin qubits have been realized in III-V semiconductors^2–4^, as well as in silicon^5–8^ and germanium^9^. The lifetime of the information encoded in such qubits is ultimately limited by the spin relaxation time, *T*_1_. This relaxation time can be estimated via transient current spectroscopy, where the excited spin state of the QD is occupied with the help of high-frequency voltage pulses, applied to one of the gates of the QD^10–13^. In single- and two-electron QDs in GaAs for example, *T*_1_ times up to 200 μs have been reported^10,11^. Group IV elements such as silicon, germanium and carbon are particularly interesting hosts for realizing spin qubits, thanks to their low nuclear spin densities and the abundance of nuclear spin free isotopes. While *T*_1_ times of up to 1 s have been reported for silicon QDs with small spin splittings^14^, *T*_1_ times of about 10 μs have been found in carbon nanotube QDs at low magnetic fields^15,16^. The latter is most likely limited by the curvature-induced spin–orbit interaction in nanotubes on the order of Δ_SO_ ≈ 1 meV^16^. In contrast, flat graphene and BLG exhibit both low hyperfine coupling and small Kane-Mele type spin–orbit interaction on the order of 40–80 μeV^17–21^, promising long spin lifetimes^22^. Early devices were based on etched QDs in single-layer graphene, where edge disorder prevented control over the charge occupation of the QDs^23–25^, imposing currently a major roadblock for single-layer graphene based qubits. In contrast, BLG is particularly suitable for realizing highly tunable QDs^26,27^, and important steps towards the realization of spin qubits have already been achieved—such as the implementation of charge detection^28,29^, the investigation of the electron-hole crossover^30^ and the measurement of the spin–orbit gap in BLG^20,21,31^. However, electrical measurements of the spin relaxation time have remained elusive in both, single-layer graphene and BLG until now^12,13^. In this letter, we report on the measurement of *T*_1_ times in a single-electron BLG QD. Our measurements confirm that the relaxation time is sufficiently long to potentially operate a spin qubit, making graphene an interesting host material for bench-marking spin qubits.

## Results

**Fig. 1:**
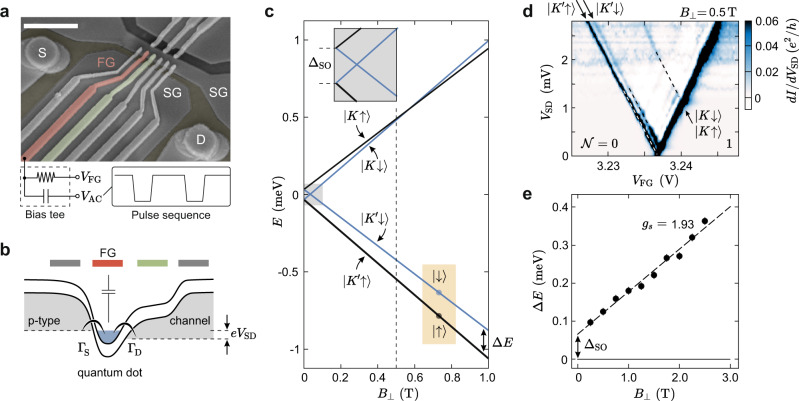
Device schematics and single-particle spectrum. **a** False-color scanning electron microscopy image of the gate layout. The SGs define a narrow conducting channel connecting source and drain, while the FGs across the channel are used to form a QD. Bias tees connected to the FGs allow the application of AC pulses (*V*_AC_) and DC voltages (*V*_FG_) to the same gate. The scale bar corresponds to 1 μm. **b** Band schematic along the channel. One FG (red) is tuned to form a QD, while the tunnel coupling to the right lead, Γ_D_, is controlled using a neighboring FG (green). **c** Single-particle spectrum of a BLG QD as function of a out-of-plane magnetic field *B*_⊥_. Inset: At *B*_⊥_ = 0 T, the spin–orbit interaction splits the four states of the first orbital into Kramer's pairs with spin–orbit gap Δ_SO_. **d** Finite bias spectroscopy measurement of the single-particle spectrum recorded at *B*_⊥_ = 0.5T (see dashed line in **c**). Dashed lines highlight the four single-particle states. **e** Measured energy splitting Δ*E* of the two $K^{\prime}$-states, $\left|\uparrow \right\rangle$ and $\left|\downarrow \right\rangle$, as a function of *B*_⊥_.

The device consists of a BLG flake encapsulated in hexagonal boron nitride (hBN) placed on a global graphite back gate (BG), with two layers of metallic top gates. Figure 1a shows a scanning electron microscopy image of the gate structure of the device (see methods for details)^30^. To form a QD, we use the BG and split gates (SG) to form a p-type channel connecting source (S) and drain (D). The potential along the channel can be controlled using a set of finger gates (FGs) and a QD is formed by locally overcompensating the potential set by the BG using one of the FGs (see red FG in Fig. 1a, b), forming a p–n–p junction, where an n-type QD is tunnel coupled to the p-type reservoirs. The electron occupation of the QD can be controlled down to the last electron using the FG potential *V*_FG_ (see Supplementary Figs. 1, 2 for details). The tunnel coupling between the QD and the channel can be tuned using adjacent FGs (e.g., green FG in Fig. 1a, b), allowing also to realize configurations with strongly asymmetric tunnel barriers, as illustrated in the schematic of Fig. 1b. All other FG potentials are kept on ground.

Figure 1c shows the energy dispersion of the first orbital state of the QD as a function of an external out-of-plane magnetic field. In BLG, each single-particle orbital is composed of four states, because of the spin and valley degrees of freedom. In contrast to silicon, the valley states in BLG are associated with topological out-of-plane magnetic moments, which originate from the finite Berry curvature close to the *K*-points and has opposite sign for the *K* and $K^{\prime}$-valley^32^. At zero magnetic field, the Kane-Mele type spin–orbit interaction^33^ splits the four degenerate states into two Kramer’s pairs $(\left|K\uparrow \right\rangle ,\left|K^{\prime} \downarrow \right\rangle )$ and $(\left|K\downarrow \right\rangle ,\left|K^{\prime} \uparrow \right\rangle )$ with an energy gap Δ_SO_^20,21^ (see inset of Fig. 1c). An out-of-plane magnetic field, *B*_⊥_, lifts the degeneracy and each state shifts in energy according to the spin and valley Zeeman effect as $E({B}_{\perp })\,=\,\frac{1}{2}(\pm {g}_{{{{{{{{\rm{s}}}}}}}}}\,\pm\, {g}_{\nu }){\mu }_{{{{{{{{\rm{B}}}}}}}}}{B}_{\perp }$, with the Bohr magneton *μ*_B_, the spin *g*-factor *g*_s_ = 2 and the valley *g*-factor *g*_*ν*_. Considering typical values of Δ_SO_ ≈ 65 μeV and *g*_*ν*_ ≈ 30^20^, valley polarization of the two lowest energy states is achieved already at about 50 mT. In this regime, the system can be treated as an effective two-level spin system with the ground state $\left|K^{\prime} \uparrow \right\rangle$ and excited state $\left|K^{\prime} \downarrow \right\rangle$ which are split by Δ*E*(*B*_⊥_) = Δ_SO_ + *g*_s_*μ*_B_*B*_⊥_.

The single-particle spectrum of the QD can be resolved by finite bias spectroscopy measurements of the ${{{{{{{\mathcal{N}}}}}}}}\,=\,0\,\to\, 1$ electron transition (Fig. 1d). At finite magnetic field, the two energetically lower $K^{\prime}$ valley polarized spin states as well as the nearly degenerate *K*-states can be well observed (see arrows and dashed lines in Fig. 1d). Figure 1e shows the extracted splitting Δ*E* of the two spin states in the $K^{\prime}$-valley, from now on denoted as $\left|\downarrow \right\rangle$ and $\left|\uparrow \right\rangle$, as a function of *B*_⊥_. From the slope we determine Δ_SO_ = 66 ± 8 μeV and *g*_s_ = 1.93 ± 0.09, which is in good agreement with earlier experiments^20,21^.

**Fig. 2:**
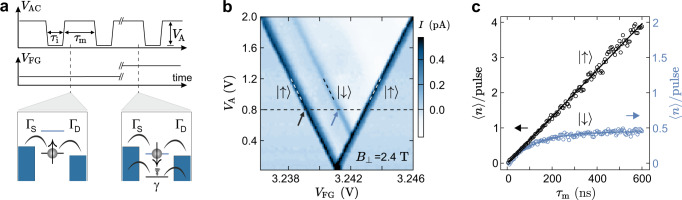
Transient current spectroscopy. **a** The schematic depicts a square pulse with amplitude *V*_A_ and pulse widths *τ*_i_ and *τ*_m_. Bottom: possible processes if the GS (left) or ES (right) reside in the bias window, which depends on the DC gate voltage, *V*_FG_. **b** Current through the QD as a function of the *V*_FG_ and the pulse amplitude *V*_A_ at the transition from ${{{{{{{\mathcal{N}}}}}}}}\,=\,0\,\to\, 1$ electrons (*V*_SD_ = 80 μV, *f* = 2.5 MHz, *τ*_m_ = *τ*_i_, *B*_⊥_ = 2.4 T). At low *V*_A_, only $\left|\uparrow \right\rangle$-transport is visible during *τ*_i_ and *τ*_m_. At *V*_A_ ≈ 0.5 V, i.e., the pulse excitation exceeding the level splitting, a transient current via $\left|\downarrow \right\rangle$ sets in. **c** Average number of electrons 〈*n*〉 per pulse cycle (〈*n*〉/pulse = *I*(*τ*_i_ + *τ*_m_)/*e*) as a function of *τ*_m_ at *τ*_i_ = 0.2 μs and *V*_A_ = 0.8 V (see black dashed line in **b**). As expected, the $\left|\uparrow \right\rangle$-transport shows a linear dependency on *τ*_m_, corresponding to a steady tunnel current (see schematic in **a**), whereas the $\left|\downarrow \right\rangle$-transport saturates due to an occupation of the ground state (see schematic in **a**). The solid line represents a fit according to $\langle n\rangle /{{{{{{{\rm{pulse}}}}}}}}\,=\,{{{\Gamma }}}_{{{{{{{{\rm{D}}}}}}}}}(1\,-\,{e}^{-\gamma {\tau }_{{{{{{{{\rm{m}}}}}}}}}})/2\gamma$.

**Fig. 3:**
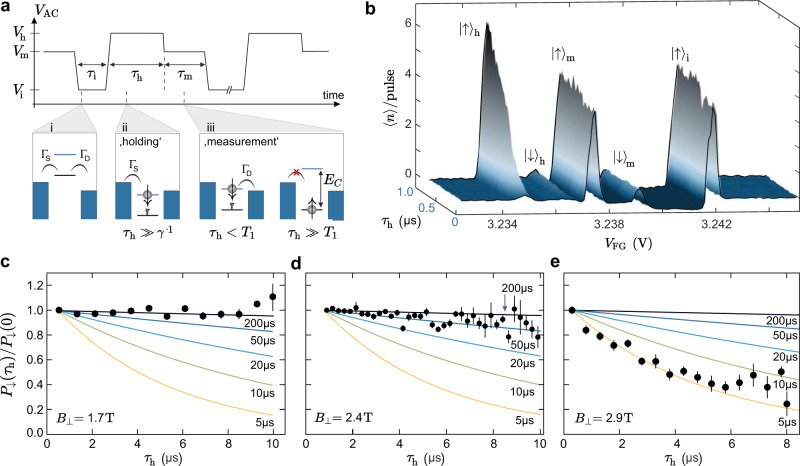
Measurement of the spin relaxation time. **a** Schematic of the applied three-level pulse train characterized by the voltages *V*_i_, *V*_h_, *V*_m_ and the times *τ*_i_, *τ*_h_, *τ*_m_. During initialization (*τ*_i_), the QD is emptied. Subsequently, both $\left|\uparrow \right\rangle$ and $\left|\downarrow \right\rangle$ are pushed below the bias window during *τ*_h_ allowing tunneling from the reservoirs into either of the states. Furthermore, relaxation from $\left|\downarrow \right\rangle$ to $\left|\uparrow \right\rangle$ is possible. In the readout step (*τ*_m_), $\left|\downarrow \right\rangle$ is aligned in the bias window, i.e., an electron in $\left|\downarrow \right\rangle$ can leave the QD contributing to the current. **b** Average number of electrons per pulse cycle 〈*n*〉/pulse = *I*(*τ*_i_ + *τ*_h_ + *τ*_m_)/*e* as a function of *V*_FG_ and *τ*_h_ (*τ*_i_ = 0.4 μs, *τ*_m_ = 0.4 μs, *V*_i_ = −1 V, *V*_h_ = 0.6 V, *V*_m_ = 0 V and *B*_⊥_ = 2.4 T). Individual line cuts of the data set are shown in Supplementary Fig. 4. **c**–**e** The probability *P*_*↓*_(*τ*_h_)/*P*_*↓*_(0) of the electron to remain in the excited state during *τ*_h_ as a function of *τ*_h_. Data has been acquired at *B*_⊥_ = 1.7, 2.4, and 2.9 T, respectively. Solid curves correspond to calculations considering different spin relaxation times *T*_1_. The error bars correspond to the current noise level in the measurement.

To gain insights on the relaxation of the $\left|\downarrow \right\rangle$ excited state to the $\left|\uparrow \right\rangle$ ground state, we now focus on transient current spectroscopy measurements. First, we use a two-level pulse scheme^11,12,34^ to extract the combined tunneling and the overall blocking rate of the system. time in BLG QDs^13^. We therefore apply a finite magnetic field of *B*_⊥_ = 2.4 T to lift the spin and valley degeneracy and, furthermore, to reduce the tunneling rates to the reservoirs^13,26^, by altering the density of states in the reservoirs^35^ and widening the tunneling barriers. Figure 2a shows the applied square pulse scheme with amplitude *V*_A_ and pulse widths *τ*_i_ and *τ*_m_. During *τ*_i_, the QD is emptied (initialized). If the ground state $\left|\uparrow \right\rangle$ is in the bias window (*eV*_SD_) during *τ*_m_, a steady current can be observed. If the excited state $\left|\downarrow \right\rangle$ is in the bias window during *τ*_m_, a transient current can be present, where electrons tunnel through the QD until one relaxes with a spin-flip or the ground state $\left|\uparrow \right\rangle$ gets occupied by direct tunneling from the reservoir. The current, *I*, through the device as a function of the pulse amplitude *V*_A_ and *V*_FG_ is shown in Fig. 2b. The two dominant transitions originate from $\left|\uparrow \right\rangle$-transport during *τ*_i_ and *τ*_m_ (see white dashed lines in Fig. 2b and left schematic in Fig. 2a). If the pulse amplitude exceeds the energy splitting of $\left|\uparrow \right\rangle$ and $\left|\downarrow \right\rangle$, a transient current can be observed during *τ*_m_ (see black dashed line in Fig. 2c and right schematic in Fig. 3a). Importantly, the rise time of the pulses needs to be faster than the inverse tunneling rates, such that the system cannot follow the pulse adiabatically (see Supplementary Fig. 3 for details).

Studying the dependence of the transient current on the pulse width *τ*_*m*_, we can extract quantitative information on the characteristic time scales of transient processes. Figure 2c shows the average number 〈*n*〉 of electrons tunneling per pulse cycle. As expected, in case of $\left|\uparrow \right\rangle$-transport, 〈*n*〉/pulse increases linearly with *τ*_m_, where the slope is given by the combined tunneling rate of both barriers Γ = Γ_S_Γ_D_/(Γ_S_ + Γ_D_) ≈ 6.6 MHz. Transport via $\left|\downarrow \right\rangle$ saturates, as the probability of blocking transport by relaxation or tunneling from the reservoir increases with *τ*_m_. To enhance transient currents, we establish an asymmetry between the source and the drain tunneling rate Γ_S_ ≫ Γ_D_ by tuning a FG adjacent to the QD. Assuming spin-independent tunnel rates, in this regime, the number of electrons tunneling via $\left|\downarrow \right\rangle$ can be approximated by $\langle n\rangle /{{{{{{{\rm{pulse}}}}}}}}\,=\,{{{\Gamma }}}_{{{{{{{{\rm{D}}}}}}}}}(1\,-\,{e}^{-\gamma {\tau }_{{{{{{{{\rm{m}}}}}}}}}})/2\gamma$, with the blocking rate *γ*^11^. A fit of the data yields a blocking rate *γ* ≈ 7.9 MHz and Γ_D_ ≈ 6.6 MHz. As *γ* is on the order of Γ_D_, direct tunneling from the reservoir into $\left|\uparrow \right\rangle$ dominates the blocking rate and, hence, the blocking rate only provides a lower bound for the relaxation time *T*_1_.

To extract *T*_1_, we then follow refs. ^10,11^ and include an additional voltage step in the pulse scheme, which allows separating the relaxation from the measurement step. The corresponding three-level pulse scheme is depicted in Fig. 3a, where the pulse segments are described by pulse durations (*τ*_i_, *τ*_h_ and *τ*_m_) and corresponding voltage values *V*_AC_ = *V*_i_, *V*_h_, and *V*_m_. During the initialization step *τ*_i_, the QD is emptied (see schematic i in Fig. 3a). Next, both states $\left|\uparrow \right\rangle$ and $\left|\downarrow \right\rangle$ are pushed below the bias window in the loading and holding step (*τ*_h_, *V*_h_). If *τ*_h_ ≫ *γ*^−1^, it is ensured that an electron has tunneled into either one of the two states (see schematic ii in Fig. 3a). Finally, to allow for spin-selective readout during the measurement step (*τ*_m_, *V*_m_), the QD levels are aligned such that only an $\left|\downarrow \right\rangle$-electron (i.e., an electron that has not relaxed) can tunnel out to the drain and contribute to the current (see schematic iii of Fig. 3a). Figure 3b shows 〈*n*〉/pulse as a function of *V*_FG_ and *τ*_h_. The three transitions labeled ${\left|\uparrow \right\rangle }_{{{{{{{{\rm{i,h,m}}}}}}}}}$ originate from $\left|\uparrow \right\rangle$ ground state transport during (*τ*_i_,*V*_i_), (*τ*_h_,*V*_h_) and (*τ*_m_,*V*_m_), respectively. As in Fig. 2c, the ${\left|\uparrow \right\rangle }_{{{{{{{{\rm{h}}}}}}}}}$ amplitude increases linearly with the duration the ground state is in the bias window, while ${\left|\downarrow \right\rangle }_{{{{{{{{\rm{h}}}}}}}}}$ saturates with the characteristic blocking rate, *γ*, of the system. The peak labeled ${\left|\downarrow \right\rangle }_{{{{{{{{\rm{m}}}}}}}}}$ originates from the electrons leaving the $\left|\downarrow \right\rangle$ excited state to the drain during the measurement step. The slight negative background between ${\left|\downarrow \right\rangle }_{{{{{{{{\rm{m}}}}}}}}}$ and ${\left|\uparrow \right\rangle }_{{{{{{{{\rm{i}}}}}}}}}$, stems from statistical backwards pumping of electrons during *τ*_i_. The relaxation time, *T*_1_, can be determined from the amplitude of the ${\left|\downarrow \right\rangle }_{{{{{{{{\rm{m}}}}}}}}}$-peak. In order to contribute to ${\left|\downarrow \right\rangle }_{{{{{{{{\rm{m}}}}}}}}}$, electrons have to remain in the excited state and not relax during *τ*_h_. The amplitude of ${\left|\downarrow \right\rangle }_{{{{{{{{\rm{m}}}}}}}}}$ as function of *τ*_h_ is directly proportional to the probability *P*_*↓*_(*τ*_h_) of an electron remaining in the excited state during *τ*_h_. Figure 3c–e show data sets for different *B*_⊥_ which have been normalized according to $\langle n({\tau }_{{{{{{{{\rm{h}}}}}}}}})\rangle /\langle n(0)\rangle \,=\,{P}_{\downarrow }({\tau }_{{{{{{{{\rm{h}}}}}}}}})/{P}_{\downarrow }(0)\,=\,{e}^{-{\tau }_{{{{{{{{\rm{h}}}}}}}}}/{T}_{1}}$ following ref. ^11^. Hence, the data is expected to follow an exponential decay, where *T*_1_ is the decay constant. The solid lines in Fig. 3c–e show the exponential decay of *P*_*↓*_(*τ*_h_)/*P*_*↓*_(0) for different values of *T*_1_. At *B*_⊥_ = 1.7 T (Fig. 3c), no decay of *P*_*↓*_(*τ*_h_)/*P*_*↓*_(0) as function of *τ*_h_ can be observed within the noise level of the data and a lower bound of *T*_1_ > 200 μs is estimated from the comparison of the data with the calculated traces. At higher magnetic fields, i.e., *B*_⊥_ = 2.4 T (see Fig. 3d) a slight and almost linear decay of *P*_*↓*_(*τ*_h_)/*P*_*↓*_(0) can be observed, which is compatible with *T*_1_ ≈ 50 μs. When further increasing the magnetic field to *B*_⊥_ = 2.9 T a clear exponential decay of *P*_*↓*_(*τ*_h_)/*P*_*↓*_(0) with *T*_1_ ≈ 5 μs can be observed (see Fig. 3e).

## Discussion

**Fig. 4:**
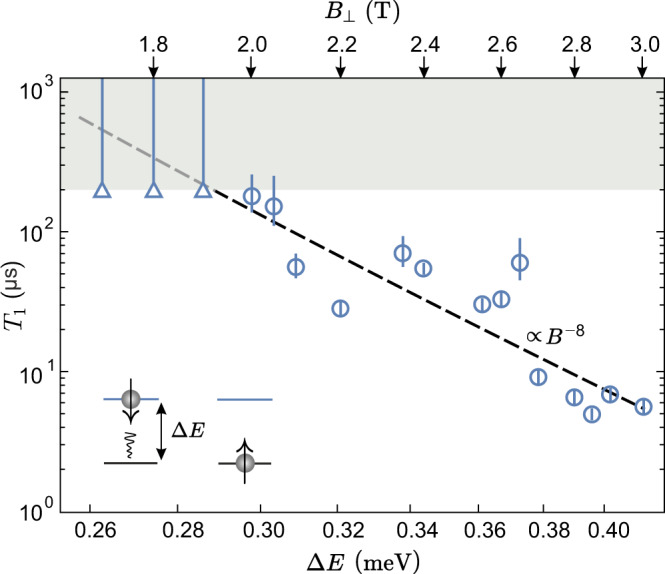
Dependence of *T*_1_ on the spin splitting. Spin relaxation time *T*_1_ as a function of the spin splitting Δ*E* = Δ_SO_ + *g*_*s*_*μ*_*B*_*B*_⊥_ and the magnetic field, *B*_⊥_, on a double logarithmic scale. The gray shaded region marks the regime where only a lower bound for *T*_1_ can be stated due to a limitation by the signal-to-noise ratio of the measurement. The dashed line marks a power law of *T*_1_ ∝ *B*^−8^. The error bars indicate the 1*σ* confidence interval of an exponential fit to the data.

Figure 4 shows *T*_1_ times extracted from exponential fits (round data points) to additional data sets as depicted in Fig. 3c–e as a function of the energy splitting Δ*E* and, hence, *B*_⊥_ (see arrows). Decreasing the magnetic field from *B*_⊥_ = 3 to 2 T, *T*_1_ increases by almost two orders of magnitude from about 5 to 200 μs. For magnetic fields below *B*_⊥_ = 2 T, no exponential decay of *P*_*↓*_(*τ*_h_)/*P*_*↓*_(0) can be fitted to the data anymore and only a lower bound of *T*_1_ > 200 μs, can be stated (see triangular data points), limited by the signal-to-noise ratio of the measured data. Upon increasing *τ*_h_ (during which no current tunnels through the QD), the average current and thus the measurement signal decreases, limiting *τ*_h_ to 10 μs, before the signal-to-noise ratio decreases below one.

Although our *B*_⊥_-field range is limited, the strong dependence of the extracted *T*_1_ times as function of the magnetic field (best described by a power law of *T*_1_ ∝ *B*^−8^, see dashed line in Fig. 4) may provide important insights on the spin relaxation mechanism. From detailed (theoretical) studies of the *B*-field dependent *T*_1_ times in GaAs QDs^36–38^, Si QDs^39–41^ and single-layer graphene nanoribbon-based QDs^42^ it is known that the spin–orbit coupling and the electron-phonon (e-ph) coupling, in particular the coupling of piezoelectric or acoustic phonons to electrons^37^ are playing a crucial role for relaxation. Indeed, it has been shown that a power-law decrease of *T*_1_ as function of increasing spin splitting Δ*E* ∝ *B* originates in such systems from enhanced phonon emission due to both, an increasing phonon density of state and an increasing (accoustic) phonon momentum with increasing Δ*E*, which in turn leads to faster spin relaxation for larger *B*-fields^37^. The spin splitting Δ*E* is composed of the Zeeman splitting, which increases linearly with *B*, as well as the constant Zeeman-like Kane-Mele spin–orbit gap, Δ_SO_ (c.f. Fig. 1e). Thus for graphene and BLG Δ*E* ∝ *B* is strictly speaking only valid if one neglects Δ_SO_. The exact exponent of the power-law scaling depends, however, sensitively on the system specific nature of (i) the e-ph coupling mechanisms, (ii) the phonons involved, (iii) the spin–orbit coupling, as well as (iv) the overall dimensionality of the system. For example, for GaAs QDs a *T*_1_ ∝ *B*^−5^ power law has been reported for *B*-fields in the range of 2–6 T^37,38^, while for small *B*-fields and suppressed spin–orbit coupling also a *T*_1_ ∝ *B*^−3^ dependence has been observed^38^. Interestingly, for Si QDs a significantly stronger power law, *T*_1_ ∝ *B*^−7^, has been predicted and observed for *B* > 2 T^39,41^, which for multidonor QDs in Si is reduced to a *T*_1_ ∝ *B*^−5^ scaling, hlighting the sensitive dependence on microscopic details. While for single-layer graphene armchair nanoribbon-based QDs an e-ph coupling dominated *T*_1_ ∝ *B*^−5^ is theoretically predicted for *B* < 3 T there is – to the best of our knowledge—no theory yet for electrostatically confined QDs in BLG. As the e-ph coupling in single-layer graphene nanoribbons and BLG are fundamentally different (just to mention the different dimensionality and the dominant gauge-field coupling in single-layer graphene^43^) it is very hard to make at the present stage any prediction of what the theoretically expected power-law dependence for BLG QDs should be. With almost certainty, electron-phonon coupling will also play an important role for BLG QDs and the observed strong *B*-field dependence of the *T*_1_ time, which gives hope for even longer times at smaller *B*-fields, may also point to a modified BLG phonon bandstructure when encapsulated in hBN. We expect that our experimental observation will trigger dedicated theoretical work on the spin relaxation in BLG QDs.

It is important to mention, that our extracted *T*_1_ times can be considered as sufficiently long for single-electron spin manipulation and mark an important step towards the implementation of spin qubits in graphene. Interestingly, the reported *T*_1_ times are more than two orders of magnitude larger than the values reported for carbon nanotubes in a similar magnetic field range^15^, most likely thanks to the smaller spin–orbit interaction in BLG. To investigate *T*_1_ times at smaller spin splittings, where spin qubits could be operated, the fabrication of devices with sufficiently opaque tunneling barriers is required, in order to achieve low tunneling rates at lower magnetic fields. Additionally, integrated charge sensors will be needed to allow for single-shot charge and spin detection.

## Methods

The device was fabricated from a BLG flake encapsulated between two hBN crystals of ~25 nm thickness using conventional van-der-Waals stacking techniques. A graphite flake is used as a BG. Cr/Au SGs with a lateral separation of 80 nm are deposited on top of the heterostructure. Isolated from the SGs by 15 nm thick atomic layer deposited Al_2_O_3_, we fabricate 70 nm wide FGs with a pitch of 150 nm.

In order to perform pulsed-gate experiments, the sample is mounted on a custom-made printed circuit board. The DC lines are low-pass-filtered (10 nF capacitors to ground). All FGs are connected to on-board bias tees, allowing for AC and DC control on the same gate. The AC lines are equipped with cryogenic attenuators of −26 dB. *V*_AC_ refers to the AC voltage applied prior to attenuation. All measurements are performed in a ^3^He/^4^He dilution refrigerator at a base temperature of around 10 mK and at an electron temperature of around 60 mK using standard DC measurement techniques. Throughout the experiment, a constant BG voltage of *V*_BG_ = −3.5 V and a SG voltage of *V*_SG_ = 1.85 V is applied to define a p-type channel between source and drain.

## Supplementary information

Supplementary Information

## Data Availability

The data supporting the findings are available in a Zenodo repository under accession code 10.5281/zenodo.6599004.
