# Supplementary material for: Spin relaxation in a single-electron graphene quantum dot
Source: Nat Commun. 2022 Jun 25;13:3637. doi: 10.1038/s41467-022-31231-5 (PMC9233672; doi:10.1038/s41467-022-31231-5)
Supplement: Supplementary file 1 — Supplementary Information [file 41467_2022_31231_MOESM1_ESM.pdf]

Supplementary Information:
Spin relaxation in a single-electron graphene quantum dot

L. Banszerus*,[1,2,*] K. Hecker*,[1,2] S. Möller,[1,2] E. Icking,[1,2]
K. Watanabe,[3] T. Taniguchi,[4] C. Volk,[1,2] and C. Stampfer[1,2]

[1] *JARA-FIT and 2nd Institute of Physics, RWTH Aachen University, 52074 Aachen, Germany, EU*

[2] *Peter Grünberg Institute (PGI-9), Forschungszentrum Jülich, 52425 Jülich, Germany, EU*

[3] *Research Center for Functional Materials, National Institute for Materials Science, 1-1 Namiki, Tsukuba 305-0044, Japan*

[4] *International Center for Materials Nanoarchitectonics, National Institute for Materials Science, 1-1 Namiki, Tsukuba 305-0044, Japan*

(Dated: May 30, 2022)

* luca.banszerus@rwth-aachen.de.

Supplementary Note 1: Formation of a single-electron quantum dot

A p-type channel is defined between the source and the drain by the voltages applied to the back gate and the split gates ($V_{BG} = -3.5$ V, $V_{SG} = 1.85$ V throughout the experiment). Fig. 1 shows the current through the channel as a function of the voltage V_{FG} applied to the finger gate (FG) (see labeling in Figs. 1a,b of the main text). Increasing V_{FG} locally compensates the potential set by the back gate and depletes the channel underneath the FG. Between $V_{FG} \approx 3.2$ V and $V_{FG} \approx 3.26$ V, the Fermi level lies in the band gap, hence the current is suppressed. A sequence of 14 Coulomb peaks can be observed starting from $V_{FG} \approx 3.26$ V indicating the formation of a quantum dot as the conduction band is pushed below the Fermi level. The peak current through the QD remains almost constant as a function of the electron occupation. Furthermore, the Coulomb resonances are grouped in quadruplets (see labels 4, 8 and 12 in Fig. S1) reflecting the fourfold shell-filling sequence, due to the spin and valley degeneracy in BLG. As the first four Coulomb resonances (as

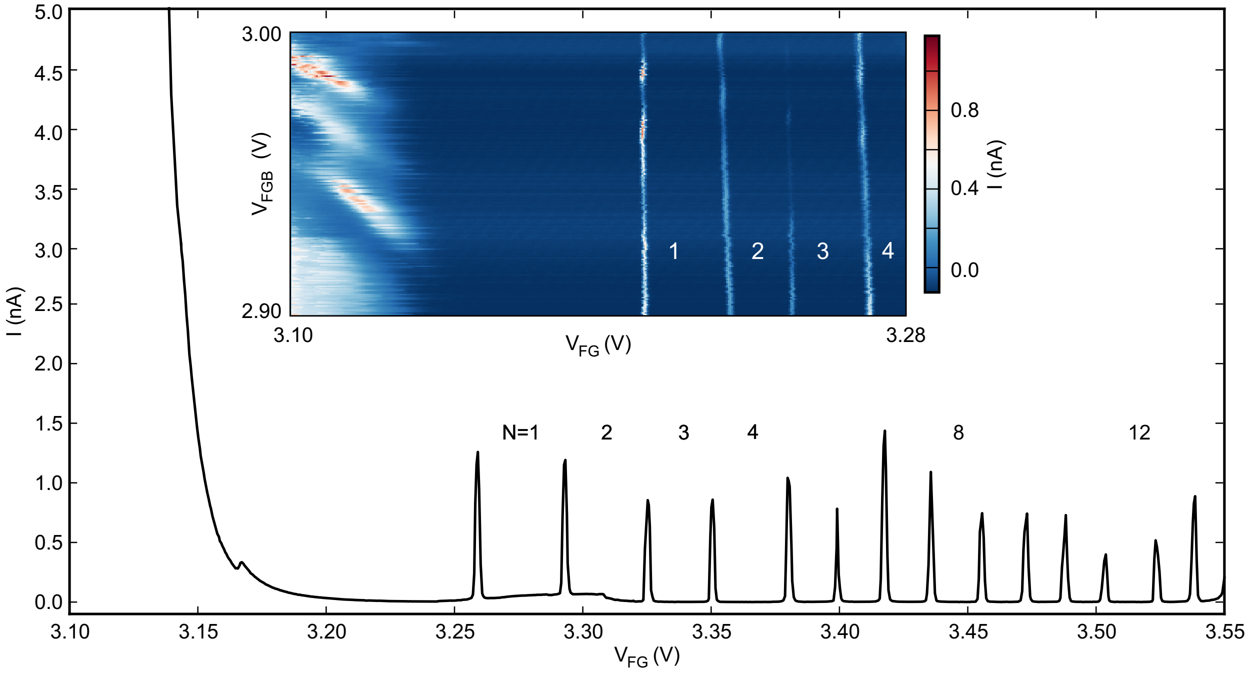

Supplementary Figure 1. Tunneling current as function of the finger gate potential V_{FG} (V_{SD} = 1 mV). Numbers indicate the electron occupation of the QD. Inset: Tunneling current as function of V_{FG} and the voltage applied to the barrier gate V_{FGB}. Note that there has been a charge rearrangement between the longer line trace and the measurement shown in the inset.

well as the next 2 × 4 peaks) are nicely grouped in four this indicates that they comprise a complete shell in agreement with the conclusion that we indeed see the 1st electron.

The inset in Fig. 1 shows a charge stability diagramm, i.e. the current as function of V_{FG} and the barrier gate voltage, V_{FGB} (see green gate in Figs. 1a,b), highlighting that the Coulomb peaks are well visible over a larger barrier gate range. With increasing V_{FGB}, the conductance decreases and the peaks shift towards lower V_{FG} due to cross capacitance effects. Please note that no additional Coulomb peak corresponding to the QD can be observed at a lower voltage than the one labeled '1', independent on V_{FGB}.

Fig. 2a shows a finite bias spectroscopy measurement around the first two Coulomb peaks at $B_{\perp} = 0$ T. At the addition of the first electron, no step in the current originating from an excited state can be observed (please compare to the 1-2 transition, where bias-symmetric

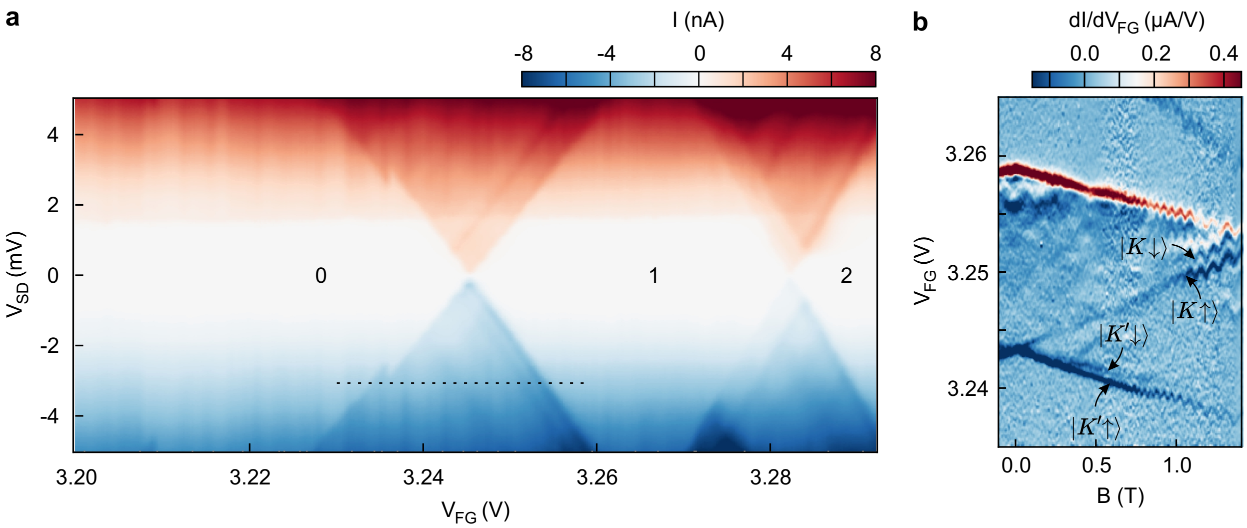

Supplementary Figure 2. **a** Tunneling current as function of the finger gate voltage, V_{FG}, and bias voltage, V_{SD}, at a magnetic field of $B_{\perp} = 0$ T. Diamond shaped region of Coulomb blockade by occupation of the first electron can be observed. **b** Transconductance measured along the dashed line in **a** as function of the perpendicular magnetic field. (Note that the absolute position of the Coulomb peak has slightly changed between measurements due to charge rearrangements in the device.) Excited states of the first electron can be observed as resonant lines in the measurement, shifting in energy due to the spin and valley Zeeman effects. Note that the oscillations at higher B-fields are due to Shubnikov de Haas oscillations in the leads (see Banszerus et al. Phys. Status Solidi B 257, 2000333 (2020)).

current steps can be observed). Peaks in the current, which are asymmetric in bias voltage, arise from resonances within the density of the states in the leads and do not depend on the excited state spectrum of the QD. Fig. 2b shows the transconductance along the dashed line in a as a function of $B = B_{\perp}$. Four states of the single-particle spectrum can be identified (see labels) which shift according to their spin and valley Zeeman effect. The measured spectrum is in perfect qualitative and quantitative agreement with the expected single-particle spectrum of a BLG QD which underlines that this transition corresponds indeed to the first electron of the QD.

Supplementary Note 2: Bandwidth of the RF coaxial lines and the pulse generator

Fig. 3a shows the transmission S_{21} of the coaxial line in the cryostate as function of frequency measured at room temperature. Fig. 3b shows an exemplary square pulse generated by the arbitrary waveform generator used in the experiment (Tektronix AWG7082C). The rise time measures ≈ 100 ps which is significantly shorter than the inverse of the tunneling rates ($\Gamma_\mathrm{S}, \Gamma_\mathrm{D}$ in the low MHz regime in our transient current spectroscopy experiments) fulfilling the condition of non-adiabaticity.

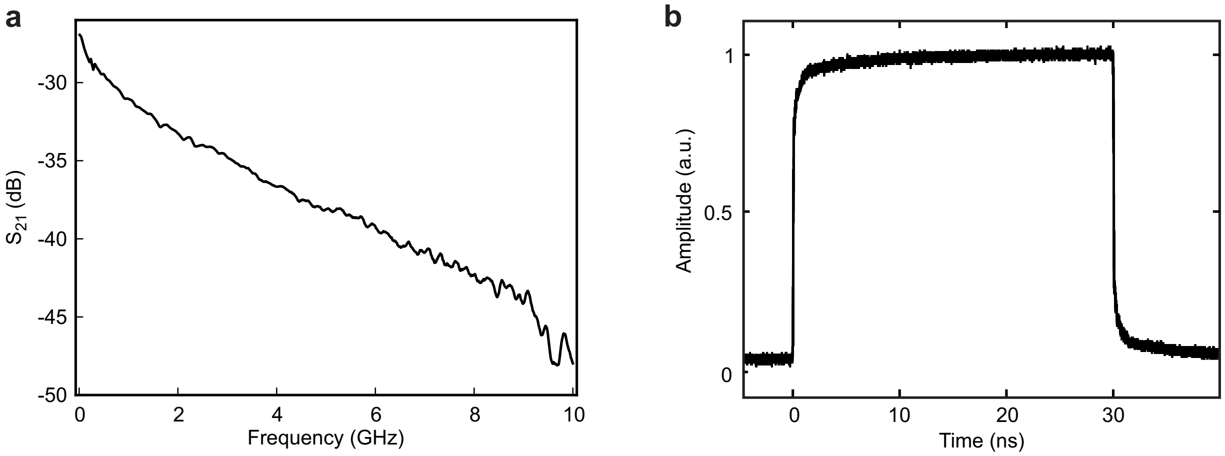

Supplementary Figure 3. **a** Transmission S_{21} of a RF coaxial line in the cryostate as function of frequency. Attenuators with a total attenuation of -26 dB are installed in the cryostate. **b** Measured square pulse generated by the AWG. The rise time of the pulse is on the order of 100 ps.

Supplementary Note 3: Complementary data to Fig. 3b

Fig. 4 shows line cuts through the data set presented in Fig. 3b of the main text. A small back pumping current is observed between $|\downarrow\rangle_m$ and $|\uparrow\rangle_i$ (around $V_{\mathrm{FG}} = 3.24$ V). Apart from that effect, the background signal is constant over the entire V_{FG} regime. To extract the $|\downarrow\rangle_m$ amplitude and thus $P_\downarrow(\tau_\mathrm{h})$, we measure the peak height relative to the background on the left side of the peak. As the background level used as reference is on the same level as the background signal far away from the QD states, we conclude that no pumping effects take place.

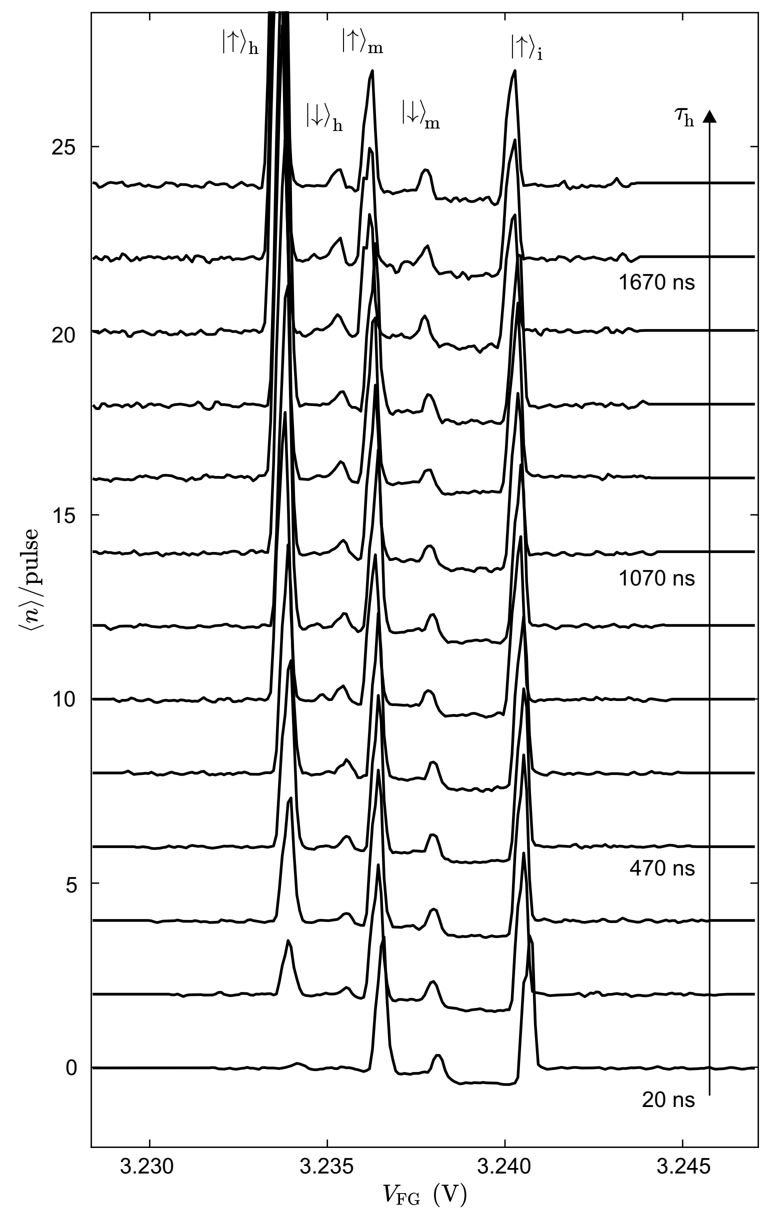

Supplementary Figure 4. Average number of electrons per pulse cycle $\langle n \rangle$/pulse as a function of V_{FG} for increasing τ_{h} (in steps of 150 ns). The traces represent cuts through the data set of Fig. 3b of the main text. Traces are offset for clarity.
